# Compact Model for Bipolar and Multilevel Resistive Switching in Metal-Oxide Memristors

**DOI:** 10.3390/mi13010098

**Published:** 2022-01-08

**Authors:** Eugeny Ryndin, Natalia Andreeva, Victor Luchinin

**Affiliations:** Department of Micro- and Nanoelectronics, Faculty of Electronics, Saint Petersburg Electrotechnical University “LETI”, 5a Professor Popov St., Building 5, 197376 Saint Petersburg, Russia; nvandr@gmail.com (N.A.); cmid_leti@mail.ru (V.L.)

**Keywords:** memristor, thin metal oxide films, compact model

## Abstract

The article presents the results of the development and study of a combined circuitry (compact) model of thin metal oxide films based memristive elements, which makes it possible to simulate both bipolar switching processes and multilevel tuning of the memristor conductivity taking into account the statistical variability of parameters for both device-to-device and cycle-to-cycle switching. The equivalent circuit of the memristive element and the equation system of the proposed model are considered. The software implementation of the model in the MATLAB has been made. The results of modeling static current-voltage characteristics and transient processes during bipolar switching and multilevel turning of the conductivity of memristive elements are obtained. A good agreement between the simulation results and the measured current-voltage characteristics of memristors based on TiO_x_ films (30 nm) and bilayer TiO_2_/Al_2_O_3_ structures (60 nm/5 nm) is demonstrated.

## 1. Introduction

Currently, all over the world, there is a significant expansion of research aimed at the development and hardware implementation of artificial neural networks (ANN), using the basic principles of the functioning of brain neurons [1,2]. The algorithms for the functioning of the ANN have a number of advantages that distinguish artificial neural networks favorably from classical computing systems based on the von Neumann architecture: the ability to learn and adapt to the working environment and process, high performance with a significant reduction in energy consumption due to asynchronous parallel data processing [3,4,5].

At present, the elements of nonvolatile resistive memory, memristors, predicted by L. Chua in 1971 [6] and for the first time manufactured in 2008 [7], are considered as the most promising candidates for the role of electronic equivalents of synapses in hardware-implemented neuromorphic systems, due to the possibility multilevel conductivity tuning, which, in combination with small topological dimensions of memristive elements (up to 2 nm [8,9]), provides a high information recording density (up to 0.7 TB/cm^2^ [10]) with low power consumption (switching energy less than 10 fJ [11]) and possibility integration into cross-bar arrays.

The physical mechanisms of nonvolatile resistive switching depend on the material of the active layer, the material of the electrodes, and the structure of the memristor. These physical mechanisms include redox reactions, phase transitions, modulation of potential barrier heights at the interface with electrodes, conductive filament formation and others [12,13,14]. Metal oxides, transition metal chalcogenides, and solid electrolytes are widely used as functional switching materials. In memristors with active layers of metal oxides, the change in conductivity is explained by the formation of a conducting filament in the active layer due to the drift of metal ions or oxygen vacancies. Recently, the possibility of nonvolatile resistive switching in various 2D materials and materials with quantum dots has been shown [12,13,14].

The main functional parameters of artificial synapses based on memristors are linearity of weight renewal, stability of the conductivity level, accuracy of weight setting, power consumption, performance, and resistance to degradation of parameters [9]. The development of memristive elements that satisfy the required values of the aforementioned parameters requires the development of appropriate physical and circuitry (compact) models.

Physical models link the electrical characteristics of memristive elements with the mechanisms of physical processes occurring in them. Such models in their initial formulation are described by systems of partial differential equations and can be realized only using numerical methods and special software [15,16,17]. The complexity of numerical implementation makes physical models inconvenient for use in the design of neuromorphic systems and in many cases limits their application to solving research problems. 

To solve the design problems of neuromorphic systems based on memristive synaptic elements, circuitry (compact) models are widely used, which imply the representation of the real structure of the memristor in the form of an equivalent circuit and are described by systems of algebraic and/or ordinary differential equations. Circuitry models are characterized by low computational complexity and usually include a certain set of fitting parameters that make it possible to sufficiently accurately fit the simulation results to specific experimental characteristics of memristive elements. These models can be integrated into SPICE-applications of modern computer-aided design (CAD) systems, supplementing their libraries of elements and expanding the functionality [18,19,20].

Numerous studies have been devoted to the development of compact models of memristive elements [18,19,20,21,22,23,24]. Usually, depending on the purpose of the study, the proposed models are designed either to simulate bipolar switching processes, or to consider multilevel tuning of the conductivity of resistive memory elements. This approach is very effective, since it makes it possible to simplify the models while maintaining an agreement between the simulation results and experimental characteristics. At the same time, when developing modern memristor based neuromorphic systems and “Logic-in-Memory” systems, it may become necessary to model memristors taking into account both bipolar switching and multilevel tuning. This is especially important for the design of cross-bar arrays of memristors based on thin oxide films, in which bipolar switching and multilevel conductivity tuning are due to fundamentally different mechanisms [8,9,10].

The purpose of this study is to develop a combined compact model of memristive elements based on thin oxide films, which makes it possible to simulate both bipolar switching processes caused by the filling and release of trap energy levels by electrons and multilevel conductivity tuning determined by the transport of oxygen vacancies in metal oxide films. The choice of memristors based on a sequence of TiO_x_ Al_2_O_3_ thin layers as the objects of modeling is due to the possibility of electric-field analog tuning of the nonvolatile resistance state in the range of seven orders of magnitude in combination with a bipolar resistive switching relatively to a given resistance state. In these structures, a 5 nm thick Al_2_O_3_ layer oxide plays a role of an active (or switching) layer, while 30–60 nm 02.thick titanium oxide layer acts as a reservoir of oxygen vacancies. Moreover, the similar devices demonstrate high switching speed, low power consumption, wide dynamic range, high resistance to cyclic degradation, high scalability, and compatibility with CMOS technologies [25,26,27,28].

The article is organized as follows: Section 2 considers the equivalent circuit of the memristive element and the equations of the proposed model; Section 3 describes a technique of memristor modeling taking into account the statistical variation of parameter values; Section 4 provides a brief description of the structure and technological process of manufacturing memristive elements based on thin films of titanium oxide TiO_x_ and on the basis of a bilayer structure TiO_2_/Al_2_O_3_, the current-voltage characteristics of which were used in this work to verify the proposed model; Section 5 discusses simulation results and their comparison with measured data; Section 6 provides general conclusions based on the results of the study.

## 2. Equivalent Circuit and Model Equations

In accordance with the results of a number of experimental and theoretical works, for example, Refs. [27,28], in memristors based on thin metal oxide films with a high density of traps, bipolar switching is determined by fast (nanosecond scale) processes of filling and releasing the energy levels of traps with electrons when a current limited by the space charge flows [15], while the multilevel conductivity tuning occurs due to much slower (millisecond scale) processes of oxygen vacancies transport.

**Figure 1:**
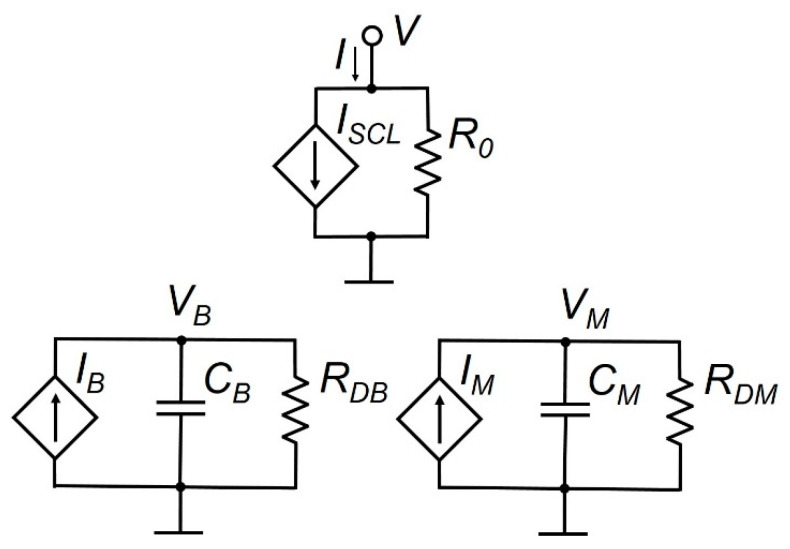
Equivalent circuit of a metal oxide memristive element.

The equivalent circuit developed taking into account the listed features of metal oxide memristive elements is shown in Figure 1.

In this equivalent circuit *V* is the voltage on the memristor contacts; *I* is the total memristor current; *I_SCL_* is the current limited by the space charge; *R*_0_ is the memristor resistance, determined by the equilibrium concentration of electrons in the dielectric film; *C_B_* is the equivalent capacitance characterizing the property of the resistive memory of the structure during bipolar switching; *V_B_* is the potential difference across the equivalent capacitance *C_B_*; *I_B_* is the voltage-controlled current source that characterizes the bipolar switching processes caused by electron transport and by processes of filling and releasing the energy levels of traps by electrons; *R_DB_* is the equivalent resistance characterizing the process of possible degradation of the memristor low-resistance state during bipolar switching; *C_M_* is the equivalent capacitance characterizing the property of the resistive memory of the structure during multilevel conductivity tuning; *V_M_* is the potential difference across the equivalent capacitance *C_M_*; *I_M_* is a voltage-controlled current source characterizing the processes of multilevel conductivity tuning caused by the transport of oxygen vacancies in the memristive structure; *R_DM_* is the equivalent resistance characterizing the relaxation process of the intermediate conductivity state of the memristor during multilevel switching. 

The elements of the equivalent circuit are described by the following system of equations:

(1) $$I=I_{SCL}+\frac{V}{R_{0}};$$

(2) $$I_{SCL}=I_{H}\left(F_{H}+F_{L}\frac{R_{OFF}}{R_{ON}}\right);$$

(3) $$I_{H}=sign\left(V\right)\frac{9}{8}\varepsilon \varepsilon_{0}\mu_{n}\frac{V^{2}}{d^{3}}S_{F}K_{M}exp\left(\frac{V_{M}}{V_{MTH}}\right);$$

(4) $$F_{H}=\frac{1}{2}-\frac{1}{\pi }arctg\left(\frac{V_{B}-\left(V_{TFLP}-V_{TFLD}\right)/2}{\varphi_{T}}\right);$$

(5) $$F_{L}=\frac{1}{2}+\frac{1}{\pi }arctg\left(\frac{V_{B}-\left(V_{TFLP}-V_{TFLD}\right)/2}{\varphi_{T}}\right);$$

(6) $$R_{0}=\frac{d}{q\mu_{n}n_{0}S};$$

(7) $$C_{B}\frac{dV_{B}}{dt}+\frac{V_{B}}{R_{DB}}=I_{B};$$

(8) $$I_{B}=sign\left(V\right)I_{FITB}F_{B}\left(V,V_{B}\right)\cdot \theta \left(\left|V\right|-\left(V_{TFLP}+V_{FITP}\right)\theta \left(V\right)+\left(V_{TFLD}+V_{FITD}\right)\theta \left(-V\right)\right);$$

(9) $$F_{B}\left(V,V_{B}\right)=exp\left(-\frac{V_{B}}{V_{BF}}\right)\theta \left(V\right)+\left[1-exp\left(-\frac{V_{B}}{V_{BF}}\right)\right]\theta \left(-V\right);$$

(10) $$C_{M}\frac{dV_{M}}{dt}+\frac{V_{M}}{R_{DM}}=I_{M};$$

(11) $$I_{M}=\frac{V}{R_{FITM}}F_{M}\left(V,V_{M}\right)\cdot \theta \left(\left|V\right|-V_{MTH}\right);$$

(12) $$F_{M}\left(V,V_{M}\right)=exp\left(-\frac{V_{M}}{V_{MP}}\right)\theta \left(V\right)+\left[1-exp\left(-\frac{V_{M}}{V_{MD}}\right)\right]\theta \left(-V\right);$$

where $\frac{R_{OFF}}{R_{ON}}$ is the ratio of the resistances of the memristor in the high-resistance and low-resistance states; q is the elementary charge; $\varphi_{T}=\frac{k_{B}T}{q}$ is the temperature potential; $k_{B}$ is Boltzmann’s constant; T is absolute temperature; t is time; d is the dielectric film thickness; S is the area of the memristive element; $S_{F}$ is the cross-sectional area of the current filament arising in the memristive element; $n_{0}$ is the equilibrium concentration of electrons in the dielectric film; $\mu_{n}$ is the electron mobility in a dielectric film; ε is the relative dielectric constant of the film material; $\varepsilon_{0}$ is the dielectric constant of vacuum; $I_{H}$ is the high-resistance state current; $F_{H},F_{L}$ are smoothing functions; $V_{TFLP},V_{TFLD}$ are the threshold voltages of SET and RESET processes, respectively, for bipolar switching; $V_{MTH}$ is the threshold voltage of the multilevel tuning of the memristor conductivity; $K_{M}$ is a dimensionless fitting parameter; $V_{FITP},V_{FITD}$ are the fitting parameters with the dimension of voltage; $I_{FITB}$ is the fitting parameter with the dimension of current; $R_{FITM}$ is the fitting parameter with the dimension of resistance; $V_{BF}$ is a fitting parameter with a voltage dimension, which determines the inertia of bipolar switching processes; $V_{MP},V_{MD}$ are tuning parameters with the dimension of voltage, which determine the inertia of the tuning processes (increase and decrease, respectively) of the conductivity level during multilevel switching; θ is the Heaviside function.

In accordance with the equivalent circuit shown in Figure 1 and expression (1), the total memristor current *I* is determined by the sum of two current components: the current $\frac{V}{R_{0}}$, determined by the equilibrium electron concentration in the dielectric film, and the current *I_SCL_*, limited by the space charge. The memristor resistance, determined by the equilibrium electron concentration in the dielectric film, is given by expression (6) [15].

The *I_SCL_* current in accordance with expression (2), depending on the state of the memristor, is determined either by the current in the high-resistance state *I_H_*, or by the current in the low-resistance state $I_{H}\frac{R_{OFF}}{R_{ON}}$, the smooth transitions between which in the process of bipolar switching are provided by smoothing functions $F_{H},F_{L}$, specified by expressions (4) and (5).

The high-resistance state current IH is determined by the trap quadratic law: $sign\left(V\right)\frac{9}{8}\varepsilon \varepsilon_{0}\mu_{n}\frac{V^{2}}{d^{3}}S_{F}$ [15]. In order to simulate both the processes of bipolar switching and multilevel tuning of the memristor conductivity, this quadratic dependence in expression (3) is supplemented by the factor $K_{M}exp\left(\frac{V_{M}}{V_{MTH}}\right)$, which determines the conductivity level of the memristor structure in the process of multilevel tuning. The value of this multiplier at the current time is determined by the change in voltage $V_{M}$ across the equivalent capacitance $C_{M}$ during its recharge with current $I_{M}$ (Figure 1). 

The effect of resistive memory during bipolar switching, in accordance with Figure 1, is described by the equivalent capacitance *C_B_* controlled by the current source *I_B_* and the equivalent resistance *R_DB_*, which characterizes the process of degradation of the low-resistance state of the memristor during bipolar switching. The change in the memristor conductivity during bipolar switching is described by differential equation (7), in which the recharge current $I_{B}$ of the equivalent capacitance $C_{B}$ is given by expressions (8) and (9). The direction of the current $I_{B}$ in accordance with expression (8) is determined by the sign of the voltage sign(V) applied to the memristor contacts. In this case, the Heaviside function $\theta \left(\left|V\right|-\left(V_{TFLP}+V_{FITP}\right)\theta \left(V\right)+\left(V_{TFLD}+V_{FITD}\right)\theta \left(-V\right)\right)$ zeroes the current $I_{B}$ if the applied voltage V is in the range

(13) $$V_{TFLD}+V_{FITD}<V<V_{TFLP}+V_{FITP}.$$

As a result, when the voltage *V* changes in the range (13), the resistive state of the memristor remains unchanged. 

The usage of smoothing functions $F_{H},F_{L}$, specified by expressions (4), (5), leads to a certain deviation of the bipolar switching thresholds on the calculated current-voltage characteristics of memristors, relatively the specified values of threshold voltages $V_{TFLP},V_{TFLD}$ of SET and RESET processes. Fitting parameters $V_{FITP},V_{FITD}$ provide compensation for the specified deviations.

The function $F_{B}\left(V,V_{B}\right)$, used in expression (8) and specified by expression (9), has a range of [0, 1] and determines limitations of the electric current $I_{B}$ by asymptotes specified by the fitting parameter $I_{FITB}$. Since the dynamics of the voltage change $V_{B}$ across the equivalent capacitance $C_{B}$ is determined in accordance with Equation (7) by the level of current $I_{B}$ and the degradation time constant of the low-resistance state of the memristor $C_{B}R_{DB}$, the fitting parameter $V_{BF}$ in expression (9) actually determines the inertia of bipolar switching processes.

The effect of resistive memory during multilevel tuning of the memristor conductivity, which is determined by a change in the spatial distribution of the concentration of oxygen vacancies in the dielectric film, in accordance with Figure 1, is described by the equivalent capacitance *C_M_*, voltage-controlled current source *I_M_*, and the equivalent resistance *R_DM_*, which characterizes the process of degradation of the memristor state during multilevel switching. The change in the memristor conductivity in the process of multilevel tuning is described by differential equation (10), in which the recharge current *I_M_* of the equivalent capacitance *C_M_* is given by expression (11). The direction and value of the current *I_M_* are determined by the voltage *V* applied to the memristor contacts and the equivalent fitting resistance *R_FITM_*. In this case, the Heaviside function $\theta \left(\left|V\right|-V_{MTH}\right)$ zeroes the current *I_M_* if the applied voltage *V* is in the range

(14) $$-V_{MTH}<V<V_{MTH}.$$

As a result, when the voltage V changes in the range (14), the multilevel tuning of the memristor conductivity does not occur. 

The function $F_{M}\left(V,V_{M}\right)$, specified by expression (12), is similar to the function $F_{B}\left(V,V_{B}\right)$ and determines the inertia of the processes of multilevel tuning of the memristor conductivity by the values of the fitting parameters $V_{MP},V_{MD}$ for conductivity increasing and decreasing respectively.

## 3. Statistical Variation of Model Parameters

In [20], the importance of taking into account the statistical variability of the main parameters of memristive elements to increase the reliability of modeling results and the efficiency of designing neuromorphic systems based on them is substantiated in detail. Moreover, it is important to take into account both the device-to-device and cycle-to-cycle variability of the parameter values. In particular, the cycle-to-cycle variability is due to the variability of filament configuration: in each cycle of bipolar switching of the memristive element, a new filament is formed, the parameters of which, with a certain degree of probability, will differ relative to the filament of the previous cycle. 

Following the conclusions of [20], the main parameters of the model (1)–(12) were set taking into account the statistical variability of their values in accordance with the expression:

(15) $$P=M\left(P\right)\left(1+\gamma D_{P}\right),$$

where P is the value of the model parameter, taking into account the statistical variability; M(P) is the mean value of the parameter P; γ is a random number corresponding to the standard normal distribution; $D_{P}$ is the relative change in the parameter value P.

When verifying the model, we used the following parameters as the parameters whose values varied in accordance with expression (15): the threshold voltages of SET and RESET processes during bipolar switching $V_{TFLP},V_{TFLD}$, the cross-sectional area of the current cord $S_{F}$, and the memristor resistance ratio in high resistance and low resistance states $\frac{R_{OFF}}{R_{ON}}$. If necessary, in each specific case of application of the proposed model, the list of randomly varied parameters can be extended.

## 4. Materials and Methods

**Figure 2:**
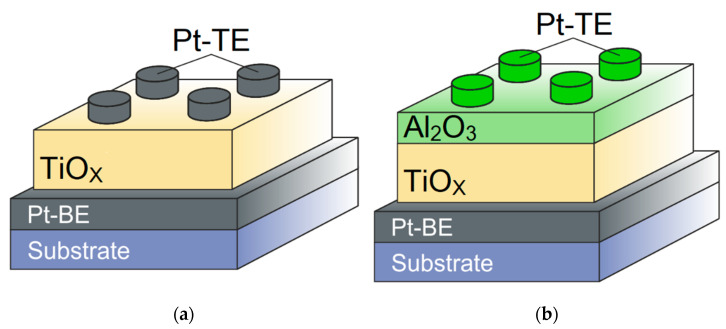
Structures of the memristors based on TiO_x_ (**a**) and TiO_2_/Al_2_O_3_ (**b**) films.

In order to validate the developed model (1)–(12), the simulation results were compared with the measured *I–V* characteristics of memristive elements based on a thin titanium oxide film TiO_x_ (bipolar switching) as well as on the basis of a bilayer film TiO_2_/Al_2_O_3_ (multilevel conductivity tuning with bipolar switching), with structures shown schematically in Figure 2.

In the process of manufacturing memristive elements with the structure shown in Figure 2a, bottom platinum electrodes (Pt-BE 50 nm) were deposited by magnetron sputtering at a temperature of T = 150 °C on a p-Si/SiO_2_ substrate with a 10 nm thick titanium adhesive layer. A titanium oxide layer TiO_x_ (30 nm) was grown on the Pt-BE surface in an atomic layer deposition equipment TFS 200 (Beneq) at a temperature of T = 150 °C using titanium isopropoxide (Ti[OCH(CH_3_)_2_]_4_) as a precursor and vapor water as an oxidizing agent. The values of the thickness of the grown layers were monitored in the course of scanning electron microscopy over a cross section of the structure formed by a focused ion beam on an FEI, Helios NanoLab system. The surface of the TiO_x_ layer was examined using an atomic force microscope Dimension 3100, Veeco. Postdeposition annealing was performed in air for 30 s at a temperature of T = 150 °C. Top platinum electrodes (Pt-TE 50 nm) were deposited by magnetron sputtering at a temperature of T = 150 °C. The diameter of the upper electrodes was 350 μm.

In the process of manufacturing memristive elements with the structure shown in Figure 2b, bottom platinum electrodes (Pt-BE 100 nm) were deposited by magnetron sputtering at a temperature of T = 150 °C on a p-Si/SiO_2_ substrate with a titanium adhesive layer (10 nm). A bilayer TiO_2_/Al_2_O_3_ structure (60 nm/5 nm) was grown on the Pt-BE surface in an atomic layer deposition system TFS 200 (Beneq) at a temperature of T = 200 °C using trimethylaluminum Al(CH_3_)_3_, tetrakis (dimethylamino) titanium C_8_H_24_N_4_Ti and water vapor. The thickness of TiO_2_ layer is increased to 60 nm in comparison with the structure shown in Figure 2a because in a bilayer TiO_2_/Al_2_O_3_ structure, the TiO_2_ layer is used as an oxygen vacancies reservoir for the active Al_2_O_3_ layer. Top platinum electrodes (Pt-TE 150 nm) were deposited by electron beam evaporation. The diameter of the upper electrodes was 100 μm. The process of fabricating the structure is described in more detail in [27].

The current-voltage characteristics of the metal oxide structures were measured using a Keithley 4200-SCS system.

## 5. Results and Discussion

In order to validate the developed model, the system of Equations (1)–(12) was solved in MATLAB using the finite difference method; in particular, Equations (7)–(12) were solved on a uniform time grid $G_{t}=\left\{t_{i}=\left(i-1\right)\Delta t|i=1,2,\ldots ,i_{MAX}\right\}$ using the following semi-implicit scheme:

(16) $$V_{B,i}=\frac{\frac{C_{B}}{\Delta t}V_{B,i-1}+sign\left(V_{i}\right)I_{FITB}F_{B,i}\theta \left(\left|V_{i}\right|-\left(V_{TFLP}+V_{FITP}\right)\theta \left(V_{i}\right)+\left(V_{TFLD}+V_{FITD}\right)\theta \left(-V_{i}\right)\right)}{\frac{C_{B}}{\Delta t}+\frac{1}{R_{DB}}};$$

(17) $$F_{B,i}=exp\left(-\frac{V_{B,i-1}}{V_{BF}}\right)\theta \left(V_{i}\right)+\left[1-exp\left(-\frac{V_{B,i-1}}{V_{BF}}\right)\right]\theta \left(-V_{i}\right);$$

(18) $$V_{M,i}=\frac{\frac{C_{M}}{\Delta t}V_{M,i-1}+\frac{V_{i}}{R_{FITM}}F_{M,i}\theta \left(\left|V_{i}\right|-V_{MTH}\right)}{\frac{C_{M}}{\Delta t}+\frac{1}{R_{DM}}};$$

(19) $$F_{M,i}=exp\left(-\frac{V_{M,i-1}}{V_{MP}}\right)\theta \left(V_{i}\right)+\left[1-exp\left(-\frac{V_{M,i-1}}{V_{MD}}\right)\right]\theta \left(-V_{i}\right),$$

where $t_{i}$ is the *i*-th point of the time grid; Δt is the step of the time grid; $V_{i},V_{B,i},F_{B,i},V_{M,i},\;F_{M,i}$ are the grid values of the corresponding functions.

**Figure 3:**
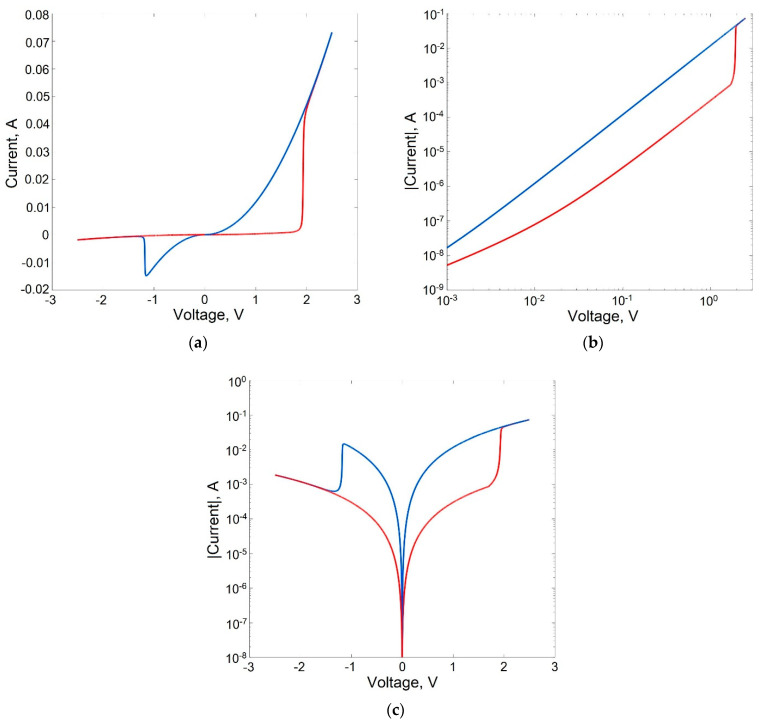
I–V characteristics of a memristive element in linear (**a**), logarithmic (**b**) and semilogarithmic (**c**) scales.

**Figure 4:**
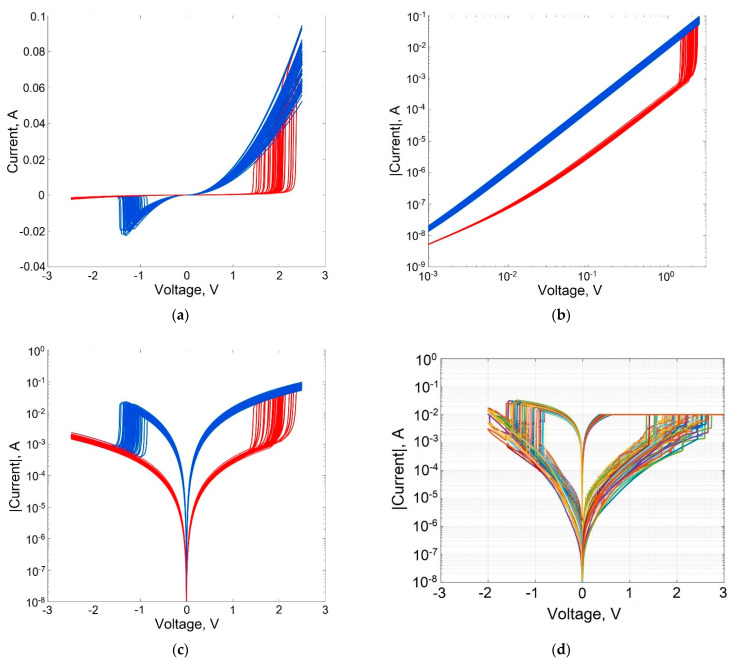
I–V characteristics of a memristive element, taking into account the statistical spread of parameters at *D_P_* = 0.1 in linear (**a**), logarithmic (**b**), and semilogarithmic scales (**c, d**): simulation results (**c**) and measurement results (**d**) for memristive structure shown in Figure 2a (measurements were carried out for 35 samples of the memristor structure).

**Figure 5:**
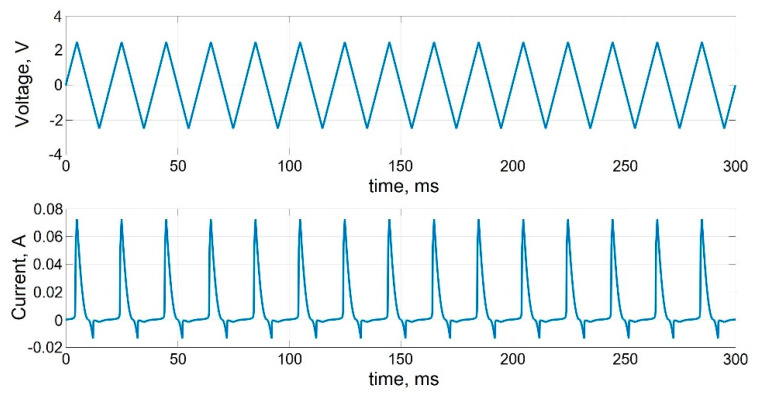
Transients of cyclic bipolar switching of memristive element.

**Figure 6:**
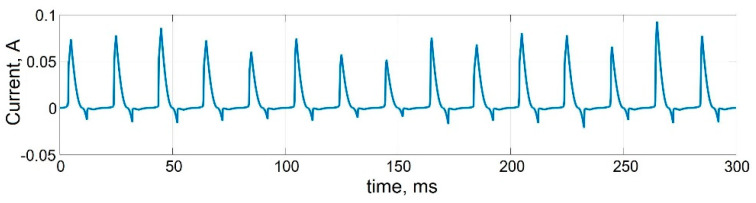
Transients of cyclic bipolar switching of memristive element taking into account the variability of parameters at *D_P_* = 0.1.

Figure 3, Figure 4, Figure 5 and Figure 6 show the results of modeling a memristive element, and the structure of which is shown in Figure 2a. The values of the memristor parameters, as well as the values of the parameters of the system (1)–(12), for which the simulation results were obtained, are given in Table 1.

Figure 3 shows the static I–V characteristics of a memristive element with bipolar switching in linear (Figure 3a), semilogarithmic (Figure 3b) and logarithmic (Figure 3c) scales, obtained from simulation without taking into account the statistical variability of the parameters ($D_{P}=0$). Red graphs correspond to the high-resistance state of the memristor and the process of switching to a low-resistance state. Blue graphs correspond to the low-resistance state and the process of switching to a high-resistance state.

The I–V characteristic plot in a logarithmic scale (Figure 3b) for the high-resistance state of the memristor (red graph) shows an ohmic (quasi-linear) section (in the voltage range *V* < 10 mV), a quadratic section (in the voltage range 10 mV–1.9 V), and a power-law (*p* > 3) switching section into a low-resistance state, corresponding to the voltage of full filling of traps with electrons $V_{TFLP}\;$= 1.9 B and a current range of 1–50 mA. After switching to a low-resistance state, the I–V characteristics become quadratic.

Figure 4 demonstrates the device-to-device variability of the static I–V characteristic of a memristive element during bipolar switching, caused by the statistical variation of parameters in accordance with the expression (15) with a relative change in the values of randomly varied parameters of the model $D_{P}\;$= 0.1 (Table 1). Comparison of the I–V characteristics of the memristor shown in Figure 4c,d indicates that taking into account the variability of the parameters of the modeled structure using a single parameter $D_{P}$ allows for achieving good agreement between the calculated data and the measurement results.

Figure 5 shows the transients of cyclic bipolar switching of a memristive element when a linearly varying voltage with an amplitude exceeding the threshold voltages $V_{TFLP},V_{TFLD}$ is applied to the memristor, without taking into account the statistical variability of the parameters. The time dependence of the current demonstrates cyclic bipolar switching of the conductivity of the memristive element. Figure 6 shows stochastic cycle-to-cycle changes of peak current values as a result of varying memristor parameters during cyclic bipolar switching at *D_P_* = 0.1.

**Figure 7:**
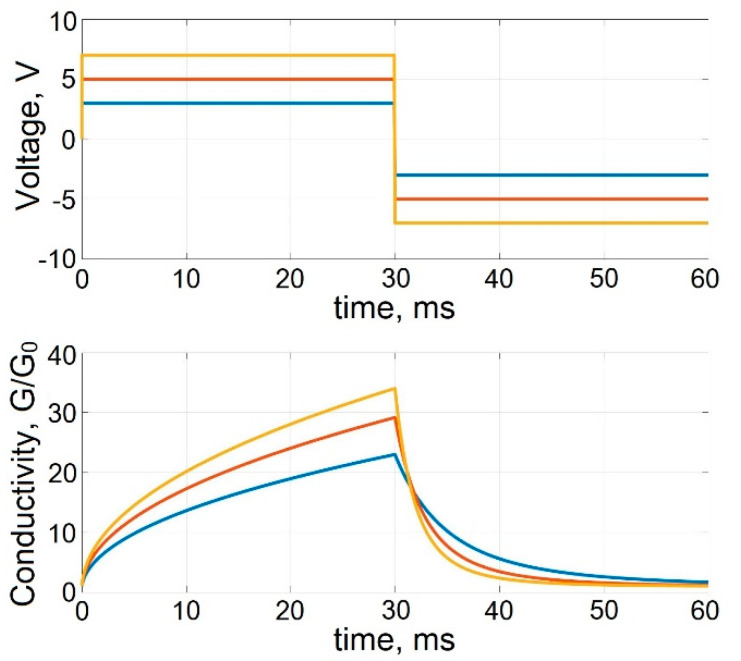
Transients of multilevel tuning of the conductivity of a memristive element.

**Figure 8:**
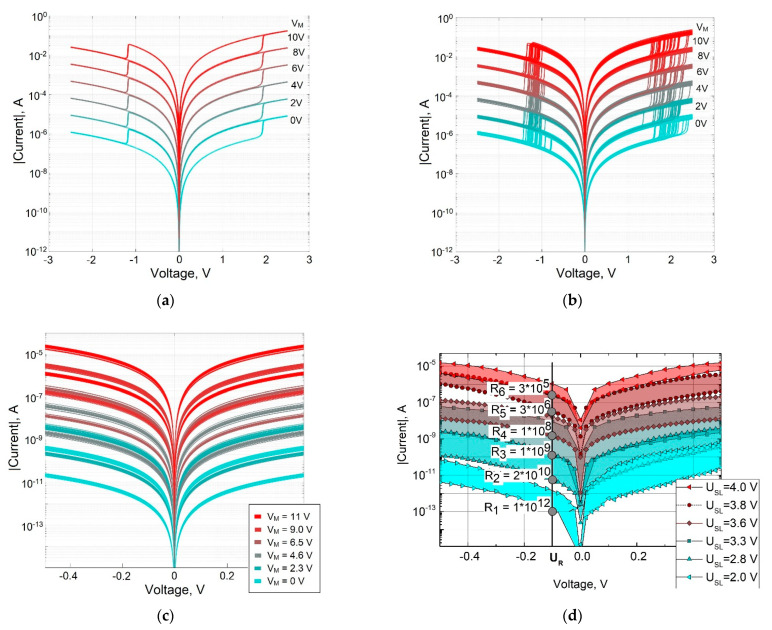
I–V characteristics of a memristive element with multilevel conductivity tuning without taking into account (**a**) and taking into account (**b**) the statistical variation of parameters, comparison of the simulation results (**c**) with the measurement data (**d**) [27] for the memristive structure shown in Figure 2b (measurements were carried out for one sample of the memristor structure).

Figure 7 and Figure 8 show the simulation results of a memristive element with the structure shown in Figure 2b. The values of the memristor parameters, as well as the values of the model parameters (1)–(12), at which the simulation results were obtained, are given in Table 2.

In accordance with the results obtained in [27], in the considered bilayer memristive structure, the characteristics of resistive memory are determined mainly by processes in the Al_2_O_3_ film: capture and release of electrons by traps (bipolar switching) and transport of oxygen vacancies (multilevel conductivity tuning). In this case, the TiO_2_ film can be considered as an unlimited source of oxygen vacancies for Al_2_O_3_ and, thus, the thickness *d_R_* of the TiO_2_ film given in Table 2 is not considered as a parameter of the model (1)–(12).

Figure 7 shows the transients of multilevel conductivity tuning of the memristive element based on bilayer TiO_2_/Al_2_O_3_ structure, calculated for bipolar voltage pulses across the memristive element with a duration of 30 ms with amplitudes of 3 V, 5 V, and 7 V, exceeding the minimum threshold *V_MTH_* of multilevel conductivity tuning. The simulation results demonstrate an increase (at *V* > 0) and, accordingly, a decrease (at *V* < 0) in the memristor conductivity level with time during the action of positive polarity pulse (in the time interval 0–30 ms) and during the action of negative polarity pulse (in the time interval 30–60 ms), as well as an increase in the rate of change in conductivity with an increase in the amplitude of the applied voltage pulses.

The static I–V characteristics of a memristive element with a combination of bipolar switching and multilevel conductivity tuning are shown in Figure 8a without taking into account the statistical variation of parameters and in Figure 8b taking into account parameter variation. For each I–V characteristic of bipolar switching, the voltage *V_M_* across the capacitance *C_M_* of the equivalent circuit (Figure 1) corresponding to the memristor conductance level during multilevel tuning is shown. 

At the same time, in accordance with the equivalent circuit and expression (3), the proposed model describes a continuous multilevel tuning of the memristor conductivity, and also, in accordance with expression (8), allows for setting different values and random variations of the threshold voltages of SET and RESET processes of bipolar switching.

In order to validate the proposed model in complex modeling of bipolar switching processes and multilevel conductivity tuning, Figure 8d shows the measured I–V characteristics of a memristor with a bilayer TiO_2_/Al_2_O_3_ structure (Figure 2b) [27], and Figure 8c shows the corresponding results of modeling this structure with parameters, given above in Table 2. Comparison of I–V characteristics in Figure 8c,d indicates sufficient agreement between the simulation results and experimental data.

## 6. Conclusions

The article presents the results of the development and study of a combined circuitry (compact) model of memristive elements made on the basis of thin metal oxide films, which makes it possible to simulate both bipolar switching processes and multilevel tuning of the memristor conductivity taking into account the statistical variability of parameters both device-to device and cycle-to-cycle.

An equivalent circuit of a memristive element and a system of equations for a compact model are given, and the parameters of the model and an equation that models their statistical variability are considered. The software implementation of the developed model in the MATLAB environment has been carried out. The results of modeling static I–V characteristics and transients during bipolar switching and multilevel tuning of the conductivity of memristive elements are obtained. A good agreement between the simulation results and the measured I–V characteristics of memristors based on TiO_x_ films (30 nm) and bilayer TiO_2_/Al_2_O_3_ structures (60 nm/5 nm) is demonstrated. 

The proposed model is quite simple to implement and can be integrated into SPICE applications of modern computer-aided design systems, supplementing their libraries and expanding the functionality.

## Figures and Tables

**Table 1:** Model parameters for a memristor with the structure shown in Figure 2a.

Parameter	Symbol	Value	Unit
Dielectric film thickness	d	3 × 10^−8^	m
Area of memristive element	S	7.07 × 10^−8^	m^2^
Cross-sectional area of the filament (mean value)	$S_{F}$	3 × 10^−16^	m^2^
Equilibrium concentration of electrons in the film	$n_{0}$	1.3 × 10^16^	m^−3^
Electron mobility	$\mu_{n}$	5 × 10^−4^	m^2^/(V*s)
Relative dielectric constant of oxide film	ε	160	–
Threshold voltage of SET process (mean value)	$V_{TFLP}$	1.9	V
Threshold voltage of RESET process (mean value)	$V_{TFLD}$	−1.2	V
Minimum threshold for multilevel switching of memristor conductance	$V_{MTH}$	2.7	V
The ratio of the resistances of a memristor in high-resistance and low-resistance states (mean value)	$\frac{R_{OFF}}{R_{ON}}$	50	–
Fitting parameter	$K_{M}$	26	–
Fitting parameter	$V_{FITP}$	−0.2	V
Fitting parameter	$V_{FITD}$	−0.2	V
Fitting parameter	$R_{FITM}$	5 × 10^8^	Ohm
Fitting parameter	$V_{BF}$	1	V
Fitting parameter	$V_{MP}$	2.5	V
Fitting parameter	$V_{MD}$	35	V
Fitting parameter	$I_{FITB}$	4 × 10^−9^	A
Elementary charge	q	1.6 × 10^−19^	C
Boltzmann constant	$k_{B}$	1.38 × 10^−23^	J/K
Dielectric constant of vacuum	$\varepsilon_{0}$	8.85 × 10^−12^	F/m
Absolute temperature	T	300	K
Relative change in the values of randomly varied parameters of the memristor model	$D_{P}$	0.1	–

**Table 2:** Model parameters for a memristor with the structure shown in Figure 2b.

Parameter	Symbol	Value	Unit
Thickness of Al_2_O_3_ dielectric film	d	5 × 10^−^^9^	m
Thickness of TiO_2_ dielectric film	$d_{R}$	6 × 10^−8^	m
Area of memristive element	S	1 × 10^−8^	m^2^
Cross-sectional area of the filament (mean value)	$S_{F}$	3 × 10^−16^	m^2^
Equilibrium concentration of electrons in the film	$n_{0}$	1.0 × 10^1^^1^	m^−3^
Electron mobility	$\mu_{n}$	5 × 10^−4^	m^2^/(V*s)
Relative dielectric constant of Al_2_O_3_ film	ε	10	–
Threshold voltage of SET process (mean value)	$V_{TFLP}$	1.5	V
Threshold voltage of RESET process (mean value)	$V_{TFLD}$	−1.5	V
Minimum threshold for multilevel switching of memristor conductance	$V_{MTH}$	2.7	V
The ratio of the resistances of a memristor in high-resistance and low-resistance states (mean value)	$\frac{R_{OFF}}{R_{ON}}$	20	–
Fitting parameter	$K_{M}$	6.7 × 10^−7^	–
Fitting parameter	$V_{FITP}$	−0.2	V
Fitting parameter	$V_{FITD}$	−0.2	V
Fitting parameter	$R_{FITM}$	1 × 10^8^	Ohm
Fitting parameter	$V_{BF}$	1	V
Fitting parameter	$V_{MP}$	1.3	V
Fitting parameter	$V_{MD}$	500	V
Fitting parameter	$I_{FITB}$	4 × 10^−9^	A
Absolute temperature	T	300	K
Relative change in the values of randomly varied parameters of the memristor model	$D_{P}$	0.1	–

## References

[1] Basheer I., Hajmeer M. (2000). Artificial Neural Networks: Fundamentals, Computing, Design, and Application. J. Microbiol. Methods.

[2] Gokmen T., Vlasov Y. (2016). Acceleration of Deep Neural Network Training with Resistive Cross-Point Devices: Design Considerations. Front. Neurosci..

[3] Kornijcuk V., Jeong D. (2019). Recent Progress in Real-Time Adaptable Digital Neuromorphic Hardware. Adv. Intell. Syst..

[4] Potok T.E., Schuman C., Young S., Patton R., Spedalieri F., Liu J., Yao K.T., Rose G., Chakma G. (2018). A Study of Complex Deep Learning Networks on High-Performance, Neuromorphic, and Quantum Computers. ACM J. Emerg. Technol. Comput. Syst..

[5] Bichler O., Querlioz D., Thorpe S.J., Bourgoin J.P., Gamrat C. (2012). Extraction of temporally correlated features from dynamic sensors with spike-timing-dependent plasticity. Neural Netw..

[6] Chua L. (1971). Memristor-the missing circuit element. IEEE Trans. Circuit Theory.

[7] Strukov D.B., Snider G.S., Stewart D.R., Williams R.S. (2008). The missing memristor found. Nature.

[8] Zhang Y., Wang Z., Zhu J., Yang Y., Rao M., Song W., Zhuo Y., Zhang X., Cui M., Shen L. (2020). Brain-Inspired Computing with Memristors: Challenges in Devices, Circuits and Systems. Appl. Phys. Rev..

[9] Xia Q., Yang J. (2019). Memristive Crossbar Arrays for Brain-Inspired Computing. Nat. Mater..

[10] Pi S., Li C., Jiang H., Xia W., Xin H., Yang J.J., Xia Q. (2019). Memristor crossbar arrays with 6-nm half-pitch and 2-nm critical dimension. Nat. Nanotechnol..

[11] Goux L., Fantini A., Kar G., Chen Y.Y., Jossart N., Degraeve R., Clima S., Govoreanu B., Lorenzo G., Pourtois G. Ultralow sub-500nA operating current high-performance TiN/Al_2_O_3_/HfO_2_/Hf/TiN bipolar RRAM achieved through understanding-based stack-engineering. Proceedings of the 2012 Symposium on VLSI Technology (VLSIT).

[12] Wang Z., Wu H., Burr G.W., Hwang C.S. (2020). Resistive switching materials for information processing. Nat. Rev. Mater..

[13] Lv Z., Wang Y., Chen J., Wang J., Zhou Y., Han S.-T. (2020). Semiconductor Quantum Dots for Memories and Neuromorphic Computing Systems. Chem. Rev..

[14] Lv Z., Xing X., Huang S., Wang Y., Chen Z., Gong Y., Zhou Y., Han S.-T. (2021). Self-assembling crystalline peptide microrod for neuromorphic function implementation. Matter.

[15] Lampert A.M., Mark P. (1970). Current Injection in Solids.

[16] Kao K.C., Hwang W. (1981). Electrical Transport in Solids.

[17] Kim S., Choi S.H., Lu W. (2014). Comprehensive Physical Model of Dynamic Resistive Switching in an Oxide Memristor. ACS Nano.

[18] Biolek Z., Biolek D., Biolkova V. (2009). SPICE Model of Memristor with Nonlinear Dopant Drift. Radioengineering.

[19] Patterson G.A., Sune J., Miranda E. (2018). SPICE simulation of memristive circuits based on memdiodes with sigmoidal threshold functions. Int. J. Circuit Theory Appl..

[20] Bengel C., Siemon A., Cüppers F., Hoffmann-Eifert S., Hardtdegen A., von Witzleben M., Hellmich L., Waser R., Menzel S. (2020). Variability-Aware Modeling of Filamentary Oxide-Based Bipolar Resistive Switching Cells Using SPICE Level Compact Models. IEEE Trans. Circuits Syst. I Regul. Pap..

[21] Xiao-Yuan W., Fitch A.L., Iu H.H.C., Sreeram V., Wei-Gui Q. (2012). Implementation of an analogue model of a memristor based on a light-dependent resistor. Chin. Phys. B.

[22] Sidhu R.K., Singh T. (2015). Different Models of Memristor. Int. J. Eng. Res. Technol..

[23] Wang Z. (2017). The Model and the Simulation of the Memristor. AIP Conf. Proc..

[24] Isah A., Nguetcho A.S.T., Binczak S., Bilbault J.-M. (2021). Comparison of the Performance of the Memristor Models in 2D Cellular Nonlinear Network. Electronics.

[25] Wong H.-S.P., Lee H.-Y., Yu S., Chen Y.-S., Wu Y., Chen P.-S., Lee B., Chen F.T., Tsai M.-J. (2012). Metal-Oxide RRAM. Proc. IEEE.

[26] Jeong D.S., Schroeder H., Breuer U., Waser R. (2008). Characteristic electroforming behavior in Pt/TiO_2_/Pt resistive switching cells depending on atmosphere. J. Appl. Phys..

[27] Andreeva N., Ivanov A., Petrov A. (2018). Multilevel resistive switching in TiO_2_/Al_2_O_3_ bilayers at low temperature. AIP Adv..

[28] Petrov A., Andreeva N., Ivanov A. (2018). Mechanism of electron transport and bipolar resistive switching in lead oxide thin films. AIP Adv..

